# Skin Improvement in Japanese Patients With Inflammatory Dermatologic Conditions Using the Injectable Hyaluronic Acid VYC‐12

**DOI:** 10.1111/jocd.70275

**Published:** 2025-06-04

**Authors:** Chiharu Watanabe

**Affiliations:** ^1^ Chiharu Dermatology Clinic Saitama Japan

**Keywords:** acne vulgaris, atopic dermatitis, fine lines, hyaluronic acid, rosacea, VYC‐12

## Abstract

**Background:**

Many skin disorders of the face have an inflammatory element, including atopic dermatitis, acne vulgaris, and rosacea. VYC‐12 is an injectable hyaluronic acid product with well‐established hydrating effects and potential anti‐inflammatory benefits.

**Aims:**

To assess the utility of VYC‐12 for improving inflammatory skin conditions.

**Methods:**

This was a single‐center, retrospective analysis of adults with a facial inflammatory skin condition that was resistant to standard treatments. A small group was also included who only required treatment for aging‐related concerns. VYC‐12 was injected into the deep dermis based on the standard technique. Imaging was used to assess skin redness and stratum corneum moisture levels. Patient satisfaction was evaluated on a five‐point Likert scale, and adverse events were recorded throughout a mean follow‐up of 4 months.

**Results:**

Fifteen patients were included (86.7% female; mean age: 40.5 ± 9.7 years). Four had atopic dermatitis, 4 had acne vulgaris, and 2 had rosacea; the other 5 had aging‐related dryness and fine lines. Patients received a mean of 3.4 ± 1.2 mL of VYC‐12. Among 10 individuals with an inflammatory skin condition, mean skin redness decreased from 57.7 (arbitrary units) at baseline to 47.9 at 4 months post‐treatment (*p* < 0.0005); mean stratum corneum moisture levels increased from 45.1 (arbitrary units) to 75.3 over this period (*p* < 0.0005). All 15 subjects were satisfied with the results. There were no adverse events.

**Conclusions:**

VYC‐12 significantly improved skin redness and hydration in patients with inflammatory skin disorders. Further studies are warranted to assess the benefits of combining VYC‐12 with standard treatments.

## 1 Introduction

A number of challenging skin conditions of the face have an underlying inflammatory element—including atopic dermatitis, acne vulgaris, and rosacea. Appropriate treatment can yield significant benefits with regard to both improving symptoms and increasing patients' self‐esteem and quality of life [1, 2, 3].

Hyaluronic acid (HA) is a naturally occurring compound that is often used in aesthetic medicine. HA injections have well‐established hydrating effects on human skin in vivo [4, 5], as well as possible effects on inflammatory markers. Although low molecular weight (MW) HA appears to be proinflammatory, high MW formulations have shown anti‐inflammatory effects in in vitro models—based on differential macrophage activation and altered cytokine expression [6, 7, 8, 9]. Furthermore, HA injections have additional putative benefits with regard to enhanced epidermal thickness, upregulation of collagens, and increased expression of regulators of tissue regeneration [7, 8, 10, 11]. Taken together, these provide a rationale for assessing the use of high‐MW HA formulations to support standard treatments for inflammatory dermatologic conditions.

VYC‐12 (Juvéderm Volite/Skinvive; Allergan Aesthetics, an AbbVie company, Irvine, CA, USA) is an injectable HA product containing 12 mg/mL of high‐MW HA. It has been approved for the treatment of superficial cutaneous depressions such as fine lines and for the improvement of skin quality attributes like hydration and elasticity. The safety and effectiveness of deep intradermal microdroplet injections of VYC‐12 in these indications were demonstrated in prospective trials [4, 5, 12, 13], as well as various real‐world analyses [14, 15, 16, 17, 18]. A high level of patient satisfaction with outcomes was also observed [12, 13].

However, there are few reports on the impact of VYC‐12 injection on inflammatory skin disorders. Although there is preliminary evidence from an Italian study that it may be effective in the treatment of these conditions [16], the utility of VYC‐12 has not yet been analyzed in Asian patients. The purpose of the present study was to examine the effectiveness and safety of VYC‐12 injections in improving skin problems associated with atopic dermatitis, acne vulgaris, and rosacea in a group of Japanese subjects.

## 2 Materials and Methods

### 2.1 Study Overview and Patients

This was a retrospective analysis of patients treated at a single center in Japan between September 2022 and March 2023.

Eligible participants were adults with facial atopic dermatitis, acne vulgaris (with or without associated scarring), or rosacea. All had been previously managed for at least 6 months according to appropriate guidelines from the Japanese Dermatological Association, and only those showing resistance to standard treatments were eligible for the study. A small group of individuals was also included who received VYC‐12 injections only for aging‐related issues such as skin dryness and fine lines, as per the product label.

Patients were excluded if they had received prior facial treatment with dermal fillers, mesotherapy, or other cosmetic procedures (e.g., laser) within the past 12 months—or were planning to do so imminently. Pregnant or lactating women were also ineligible. Drugs known to increase coagulation time were withdrawn for 10 days before and 3 days after treatment.

The study was performed in accordance with the Declaration of Helsinki, and all enrolled subjects provided written informed consent for publication prior to treatment. In Japan, there is no requirement for investigators to have the protocol of case series with a small sample size reviewed by an Institutional Review Board.

### 2.2 Techniques

VYC‐12 was injected into the deep dermal layer of the facial skin using the syringe and 32G ½″ (1.27 cm) needle provided with the product. Injection points were equally spaced—around 1 cm apart—to facilitate uniform distribution, with 0.01–0.025 mL of product administered in each. Overall product volumes were typically 2 mL for the cheeks alone or 4 mL for the entire face, although there was scope for customization based on clinical needs. All treatments were completed in a single session with no touch‐ups.

Particular care was taken to ensure that injections were not made overly deep, thereby reducing the likelihood of bleeding. In high‐risk areas, aspiration was performed before product deposition in order to minimize the possibility of intravascular placement. If any bumps or blebs were visible post‐injection, the skin was thoroughly massaged until these were flattened.

### 2.3 Assessments

Skin quality was measured over time using two separate instruments. VISIA (Canfield Scientific, Parsippany, NJ) was utilized for the assessment of stratum corneum moisture levels (in arbitrary units) and the number of wrinkles; and ANTERA 3D (Miravex, Dublin, Ireland) was used for assessing skin redness (hemoglobin concentration; arbitrary units).

All patients were asked to evaluate their satisfaction with outcomes at 3 months post‐treatment, using a five‐point Likert scale (very satisfied; somewhat satisfied; neither satisfied nor dissatisfied; somewhat dissatisfied; very dissatisfied).

Adverse events were recorded throughout a mean follow‐up of 4 months.

### 2.4 Statistical Analyses

Descriptive statistics are provided throughout the paper, including mean and standard deviation for continuous variables, and frequency and percentage for categorical variables. Paired *t*‐tests were used to compare assessments of stratum corneum moisture levels and skin redness at baseline and 4 months post‐treatment. A *p* value < 0.05 was considered to be statistically significant.

## 3 Results

A total of 15 patients were included (Table 1). Thirteen (86.7%) were female and the mean age was 40.5 ± 9.7 years (range: 21–53 years). Four were treated for atopic dermatitis, 4 for acne vulgaris (2 with associated scarring and 2 without), and 2 for rosacea; the remaining 5 individuals were treated for aging‐related concerns (dryness and fine lines). They received a mean of 3.4 ± 1.2 mL of VYC‐12 (range: 2–6 mL).

**TABLE 1:** Patient characteristics.

Characteristic	Patients (*N* = 15)
Sex, *n* (%)	
Female	13 (86.7)
Male	2 (13.3)
Age, mean (SD)	40.5 (9.7)
Treatment indication, *n* (%)	
Atopic dermatitis	4 (26.7)
Acne vulgaris	4 (26.7)
Rosacea	2 (13.3)
Aging (dryness/fine lines)	5 (33.3)
Volume of VYC‐12 injected, mL, mean (SD)	3.4 (1.2)

Abbreviation: SD, standard deviation.

**FIGURE 1:**
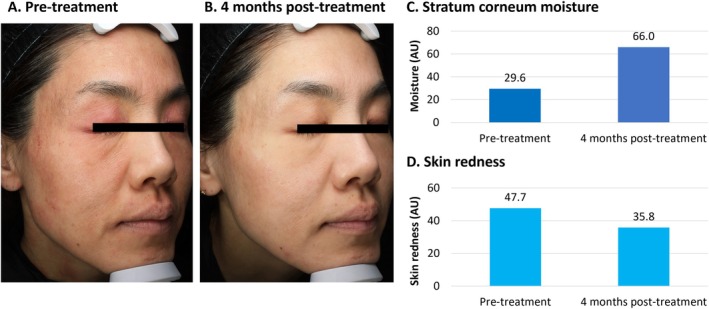
Treatment of atopic dermatitis with VYC‐12. This was a 47‐year‐old female with atopic dermatitis and a history of allergic rhinitis. She had experienced erythema and itching on the neck, arms, hands, and face for around 20 years, and presented with both erythema and scaling of the face (A). She had previously been treated using antihistamines and strong topical steroids. She was injected with 4 mL of VYC‐12 across the entire face, including the eye area but excluding the nose. No topical steroids were used post‐injection. At 4 months, her condition was improved, and wrinkles around the eyes were also decreased (B). Objective assessments demonstrated increased moisture content in the stratum corneum using Visia (C) and decreased skin redness using Antera 3D (D). AU, arbitrary units.

**FIGURE 2:**
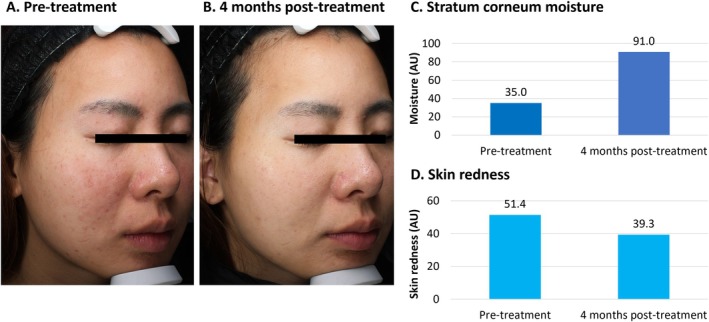
Treatment of acne scarring using VYC‐12. This was a 25‐year‐old female with acne vulgaris and acne scarring. She had a history of allergic rhinitis. The patient had experienced acne vulgaris on the face for several years, which tended to get worse with benzoyl peroxide and adapalene gel. She had erythematous papules and bothersome acne scarring on the cheeks (A). She was treated with 2 mL of VYC‐12 injected into both cheeks and the chin. At 4 months, the acne was milder, and scarring was less noticeable (B). Objective assessments demonstrated increased moisture content in the stratum corneum using Visia (C) and decreased skin redness using Antera 3D (D). AU, arbitrary units.

**FIGURE 3:**
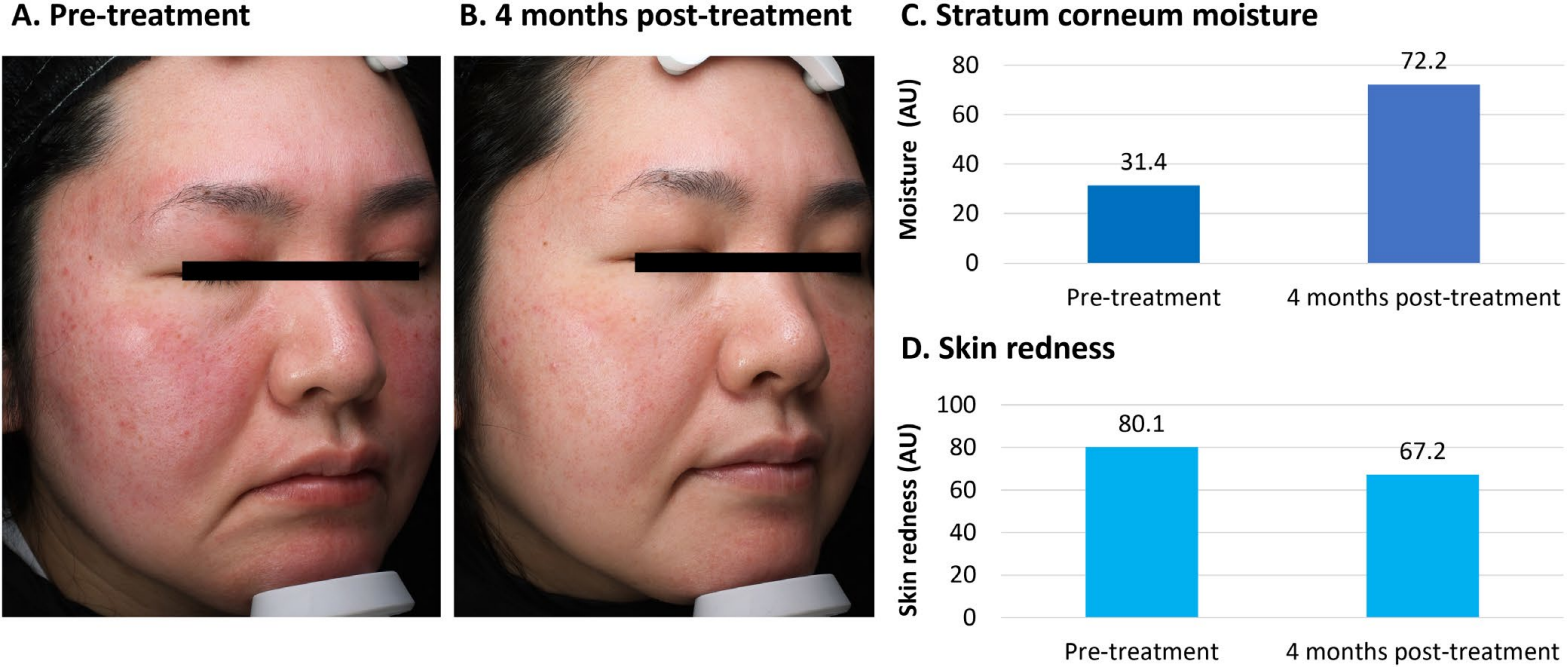
Treatment of rosacea using VYC‐12. This was a 43‐year‐old female with rosacea (II degree), and a history of urticaria. She had experienced facial redness for around 5 years. Topical applications (hydrocortisone, tacrolimus, metronidazole, and doxycycline) had not improved her condition. She presented with erythema and papules on the cheeks and chin (A). She was treated with 2 mL of VYC‐12 injected across both cheeks and the chin. At 4 months, the appearance of erythematous papules had ceased, and skin dryness was improved (B). Objective assessments demonstrated increased moisture content in the stratum corneum using Visia (C) and decreased skin redness using Antera 3D (D). AU, arbitrary units.

**FIGURE 4:**
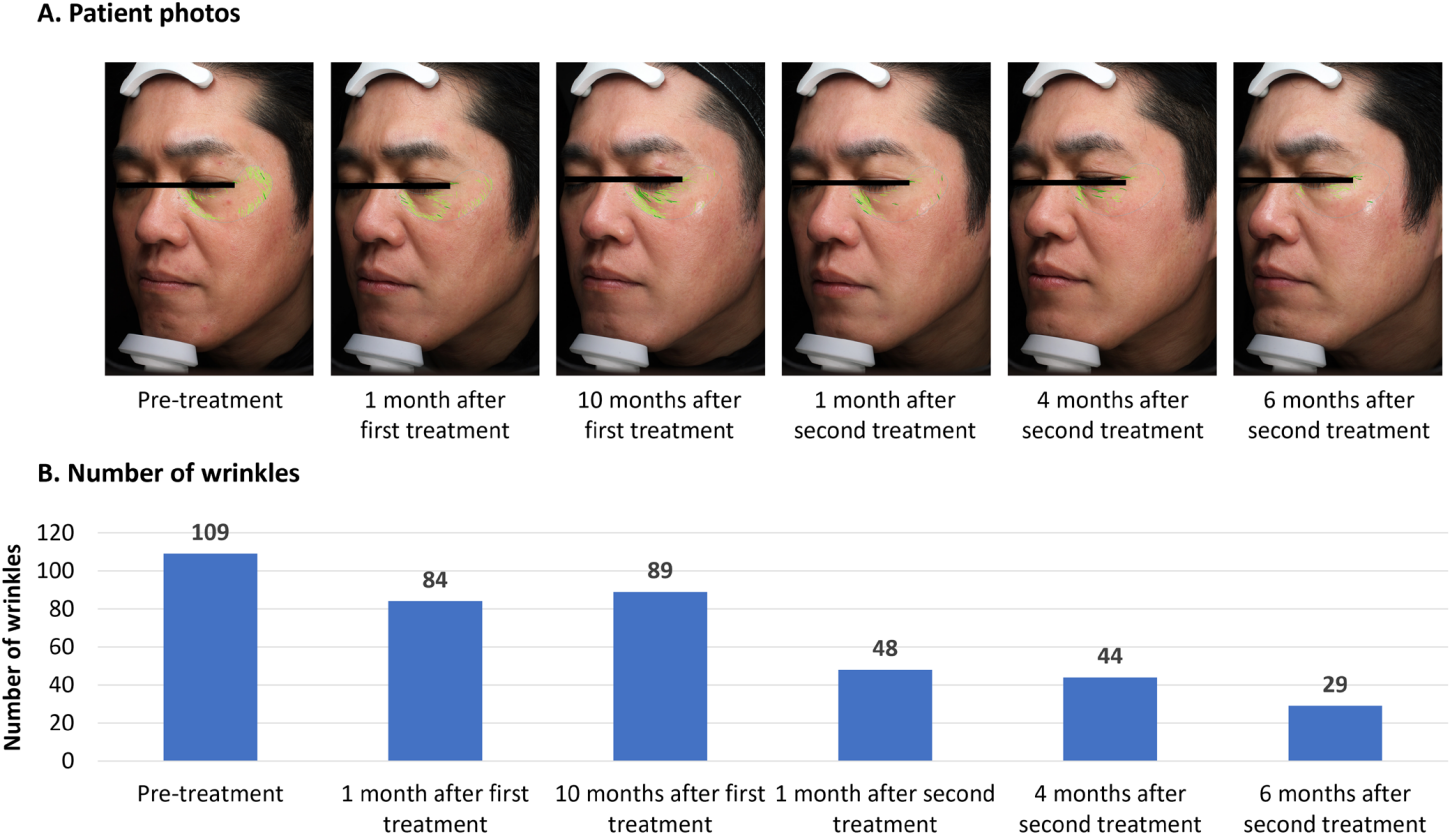
Treatment of aging‐related dryness and fine lines using VYC‐12. This was a 47‐year‐old male with skin dryness and bothersome, fine, shallow wrinkles, mainly around the eye area (A). He was treated with 5 mL of VYC‐12 injected across the whole face, except the nose. Eleven months later, he was treated again using 4 mL of VYC‐12. Over time, the dryness of the entire skin was reduced and its luster was improved (A). Objective assessment using Visia demonstrated decreasing numbers of wrinkles (B).

Example cases demonstrating the impact of treatment in each disorder are provided in Figures 1, 2, 3, 4.

**FIGURE 5:**
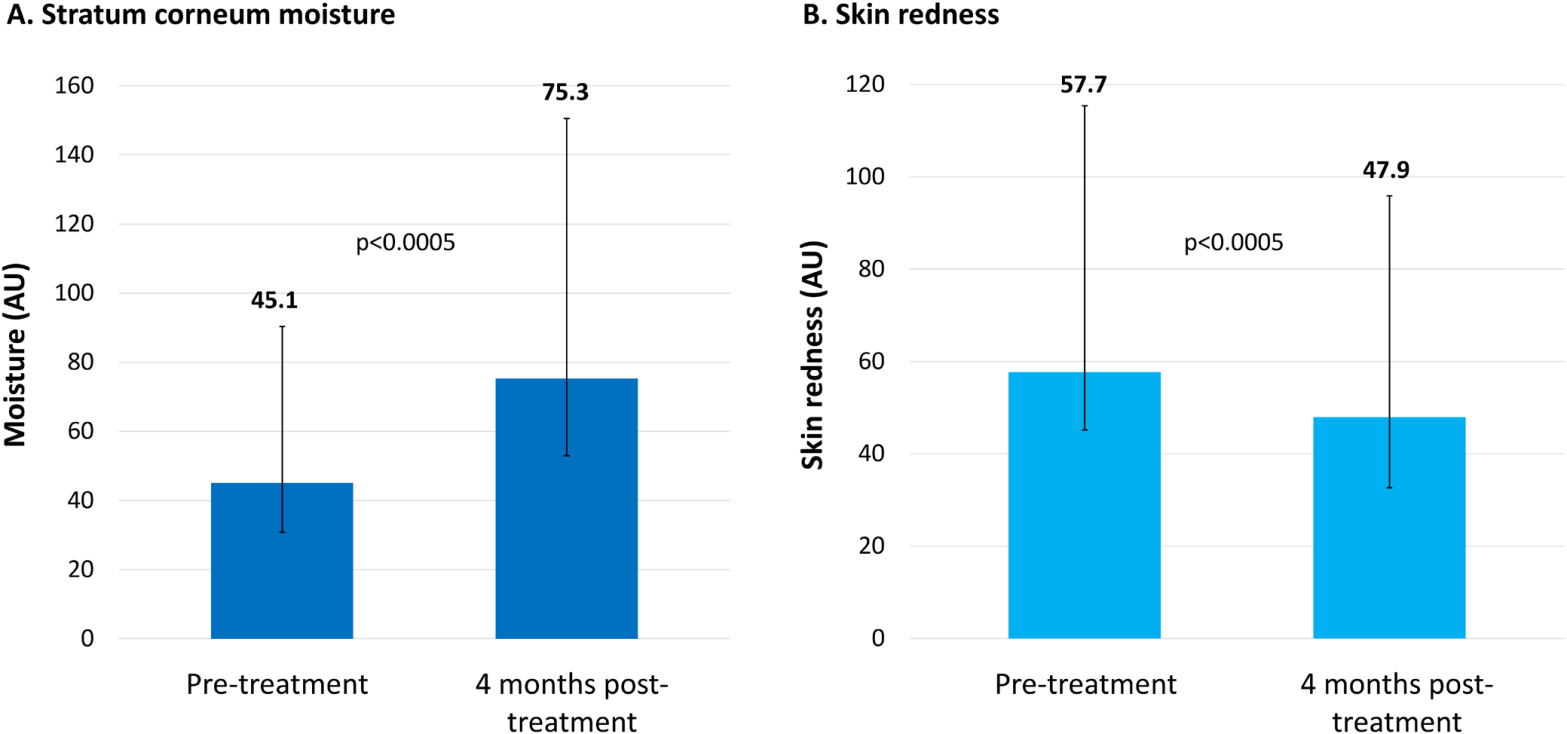
Changes in mean stratum corneum moisture levels and skin redness in patients with atopic dermatitis, acne, or rosacea. Stratum corneum moisture levels were assessed using Visia (A). Skin redness (hemoglobin concentration) was assessed using Antera 3D (B). *N* = 10. AU, arbitrary units.

Stratum corneum moisture levels were assessed objectively using the Visia technology. Among the 10 patients with atopic dermatitis, acne vulgaris, or rosacea, mean stratum corneum moisture levels increased from 45.1 (arbitrary units) at baseline to 75.3 at 4 months post‐treatment (*p* < 0.0005) (Figure 5A). Similarly, mean skin redness—assessed using Antera 3D—decreased from 57.7 (arbitrary units) before treatment to 47.9 at 4 months post‐treatment (*p* < 0.0005) (Figure 5B).

**FIGURE 6:**
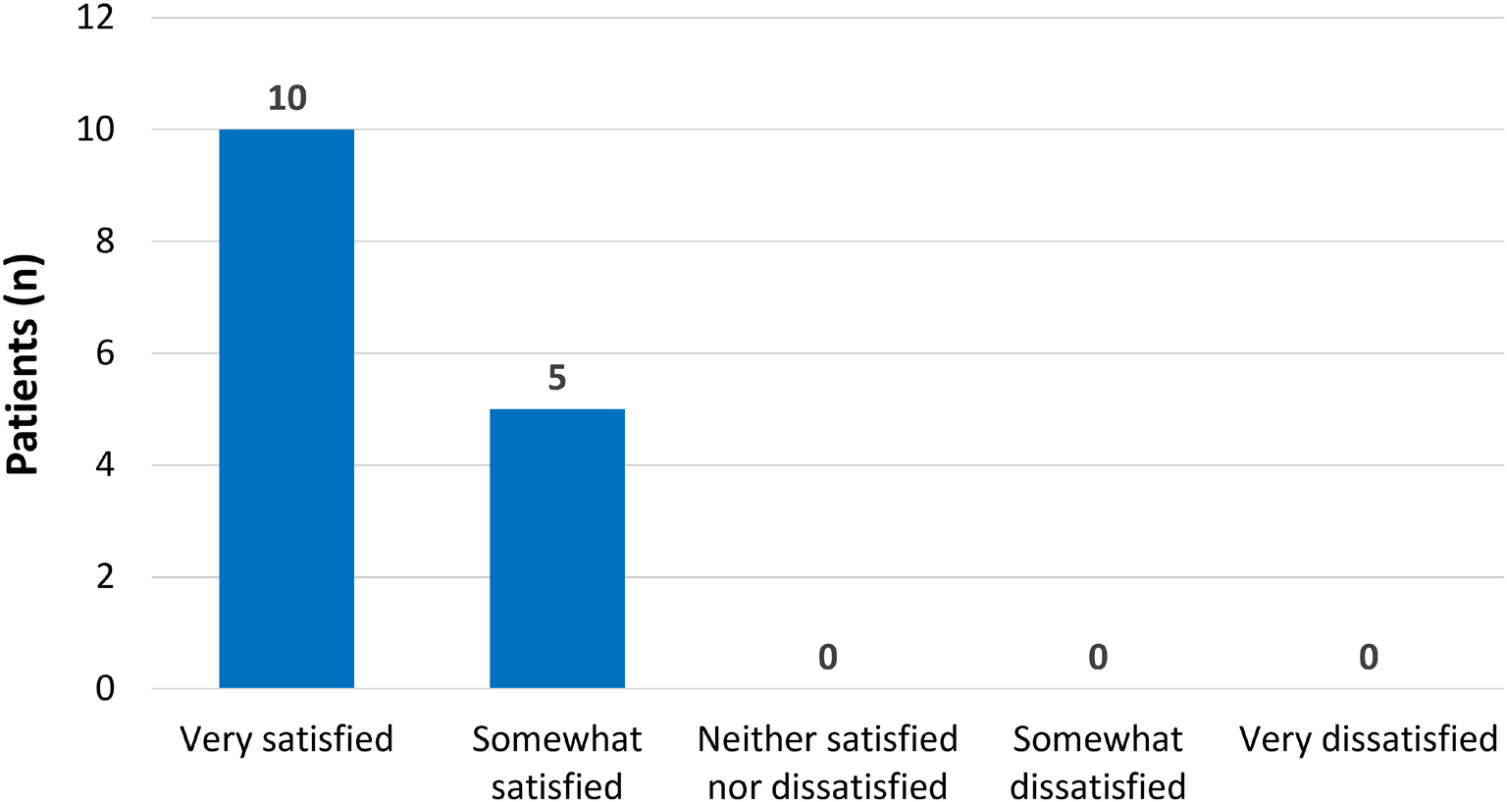
Patient satisfaction. Assessments were made at 3 months post‐treatment. *N* = 15.

All 15 study subjects were asked to assess their satisfaction with treatment at 3 months post‐injection using a five‐point Likert scale (Figure 6). Ten (66.7%) said they were “very satisfied” and the other 5 (33.3%) said they were “somewhat satisfied”.

No patients experienced any adverse events.

## 4 Discussion

In this retrospective case series, 10 Japanese patients with inflammatory dermatologic conditions (atopic dermatitis, acne vulgaris, or rosacea) were injected with the HA product, VYC‐12. Treatment led to statistically significant and clinically relevant improvements in both skin redness and hydration of the stratum corneum. The latter is associated with increased skin barrier function, and this could be a key factor underlying putative anti‐inflammatory effects of injected HA [2, 7, 19]. Positive results were also obtained in a small group of patients (*n* = 5) without an inflammatory condition, treated for skin dryness and fine lines. Regardless of the underlying disorder, before‐and‐after images show the impact at an individual level.

There are previous preliminary data from an Italian study of VYC‐12 in the treatment of inflammatory dermatologic conditions, suggesting that this approach may be effective and safe [16]. Moreover, a recent case report described improvements in acne scarring following multimodal treatment with VYC‐12 alongside a hybrid filler, laser resurfacing, and chemical peel [20]. However, to the best of my knowledge, the current study is the first to demonstrate significant improvements in objectively assessed effectiveness endpoints, and the first analysis of VYC‐12 for the treatment of inflammatory skin conditions among Asian patients. The latter is especially pertinent given the high prevalence of these disorders—particularly acne—in Asia relative to other regions [21].

The injection technique employed was based on deep intradermal microdroplet deposition with equally spaced injection points to ensure even product distribution. This aligns with the standard method described in the product label, studied in prospective trials [4, 5], and recommended in an expert consensus on VYC‐12 treatment for fine lines [22]. No specific technical alterations were required in the current group with inflammatory skin disorders. All of the study subjects were Japanese, and it is important to remember when treating Asian patients that their skin is typically thicker than among Caucasians [23]. This has important implications for optimizing injection depth into the deep dermis. Nonetheless, as noted in the expert consensus, the increased barrier function of the skin of Asian patients may help to support positive and durable outcomes with VYC‐12 [22].

Indeed, given the typically long‐term nature of these conditions, durability of effect is highly relevant in order to mitigate the need for frequent re‐treatment. Outcome duration was not assessed in the current analysis, but a European prospective study of VYC‐12 noted improvements in hydration and patient satisfaction lasting up to 9 months [4, 12].

No patients in the current dataset experienced any adverse events throughout a mean follow‐up period of 4 months. In prospective clinical trials, some individuals experienced mild short‐term sequelae, such as pain, swelling, and tenderness in the treatment area [4, 5]. Careful optimization of injection depth is integral to safe and effective outcomes, and practitioners are advised to seek adequate training before attempting these techniques. There is no specific reason to expect the safety profile of VYC‐12 to differ substantially in patients with inflammatory skin conditions compared to those without, but further study may be needed to confirm this.

Additional work is also required to fully understand the mechanistic basis for improving these disorders with injected HA. VYC‐12 injections have established hydrating effects, which were demonstrated in prospective trials [4, 5], and supported by analysis of stratum corneum moisture levels in the current paper. These effects may enhance the barrier function of the skin, helping to block the entry of allergens and bacteria into the epidermis [2]. There are also data suggesting that high‐MW HA has anti‐inflammatory effects. For example, in a murine macrophage model, it was associated with upregulation of pro‐resolving genes [6]. More recently, analyses of the effects of HA injection in human skin models found downregulation of key proinflammatory markers, such as interleukins‐36 and ‐1β and tumor necrosis factor‐alpha (TNF‐α) [7, 8]. In addition, an analysis of punch biopsies from VYC‐12‐treated human skin found upregulation of various genes involved in adipocyte differentiation, lipid metabolism, keratinocyte renewal, and maintenance of the dermal extracellular matrix [11], many of which could be relevant to the improvement of inflammatory dermatologic conditions.

There are of course important limitations to the present work, particularly the small size of the patient population, lack of a comparator treatment group, and the involvement of only a single center. Nonetheless, the data provide a foundation for further study of VYC‐12 in patients with inflammatory skin conditions, ideally in prospective, controlled trials.

## 5 Conclusion

Deep‐dermal microdroplet injection of VYC‐12 across affected areas of the face led to significant improvements in skin redness in patients with atopic dermatitis, acne vulgaris, or rosacea. It was also associated with significantly increased skin hydration and high levels of overall patient satisfaction. Additional study is warranted to further explore the benefits of combining VYC‐12 with standard treatments in patients with inflammatory dermatologic conditions.

## Disclosure

Chiharu Watanabe is an advisor of Allergan Aesthetics, an AbbVie company.

## Ethics Statement

The study was performed in accordance with the Declaration of Helsinki, and all enrolled subjects provided written informed consent for publication prior to treatment. In Japan, there is no requirement for investigators to have the protocol of case series with a small sample size reviewed by an Institutional Review Board.

## Consent

All of the patients whose photographs are used in this publication provided written informed consent.

## Conflicts of Interest

Chiharu Watanabe is an advisor of Allergan Aesthetics, an AbbVie company.

## Data Availability

The data that support the findings of this study are available from the corresponding author upon reasonable request.
